# Hyperlipidaemia treatment and gut microbiology

**DOI:** 10.3389/fmicb.2024.1520252

**Published:** 2025-01-10

**Authors:** Liu Zhe, Yu ChunLi

**Affiliations:** ^1^Shaanxi Provincial Nuclear Industry 215 Hospital, Xianyang, Shaanxi, China; ^2^The First Clinical College, Zunyi Medical University, Zunyi, Guizhou, China

**Keywords:** hyperlipidaemia, gut microbiota, gut flora, treatment, mechanisms

## Abstract

Numerous studies have shown that hyperlipidaemia is closely related to the gut microbiota, and the study of microbiota in the treatment of hyperlipidaemia is undoubtedly a new target for the treatment and prevention of hyperlipidaemia. The efficacy of regulating the gut microecology and changing the structure of gut flora has been demonstrated by both western and traditional medication, biological therapy, and dietary exercise, so it is particularly important to study the relationship between gut microbiota and the treatment of hyperlipidaemia. In this review, we summarize the mechanism and relationship between the pathogenesis of hyperlipidaemia and gut microbiota, and the mechanism of hyperlipidaemia treatment by influencing the gut microbiota in various treatment modalities, which provides diversified therapeutic ideas and scientific basis for clinical treatment. It also triggers us to think about the relationship between gut microbiota and other diseases, and to explore the influence of gut microbiota is a goal that we still need to explore.

## 1 Introduction

Hyperlipidaemia is a metabolic disease characterized by abnormal increase in total cholesterol (TC), triglycerides (TG), low-density lipoprotein cholesterol (LDL-C), and abnormal decrease in high-density lipoprotein cholesterol (HDL-C) and other indicators caused by impaired lipid metabolism in the body. Research shows that hyperlipidaemia is an important risk factor for atherosclerotic cardiovascular disease (ASCVD), which willing significantly increases the risk of cardiovascular events such as coronary artery disease, myocardial infarction, stroke and peripheral arterial disease. Meanwhile, hyperlipidaemia is thought to be the cause of 29.7 million DALYS (disability-adjusted life years) or 2% of all DALYS and 2.6 million deaths (4.5% of total deaths) (Abbasi et al., 2024). Therefore, it is particularly important to pay attention to the pathogenesis and treatment of hyperlipidaemia. The development of hyperlipidaemia is related to exogenous lipid absorption and endogenous lipid synthesis. Gut lipid synthesis and absorption play an important role in the development of hyperlipidaemia, and in recent years, gut flora have been found to be involved in lipid synthesis, absorption and catabolism. The role of gut flora in the development of hyperlipidaemia has received close attention. The gut tract is an important digestive organ of the human body, and the number of microbiota in the normal human gut tract is more than 10^14^, and there are more than 800 types of microbiota, such as bacteria, fungi, viruses and other microbiota, of which the bacteria include: Clostridium, Lactobacillus, Bifidobacterium, Bacteroides and *Escherichia coli* are the five main genera (Kreulen et al., 2023). In healthy people, gut microbiota maintain a microscopic ecological balance, which is essential for maintaining the normal physiological functioning of the human body. Research on the mechanism and treatment of hyperlipidaemia has shown that gut microbiota are inextricably linked to hyperlipidaemia. Gut microbiota can participate in the synthesis and metabolism of TG and TC and other biochemical processes through various pathways. Due to the fact that gut microbiota can improve the efficiency of food energy intake, regulate and affect lipid metabolism, and cause long-term low-level chronic inflammatory reactions, studying the relationship between gut microbiota and hyperlipidaemia is of great significance. Therefore, this article will review the mechanism and treatment of hyperlipidaemia from the perspective of gut microbiota. In this review, we summarized the relevant mechanisms of gut microbiota in the pathogenesis of hyperlipidaemia, as well as the roles and mechanisms of gut microbiota production in various treatment methods for hyperlipidaemia. Our aim is to provide a diversified and comprehensive reference for the clinical treatment of hyperlipidaemia, while also providing a scientific basis for the treatment and prevention of hyperlipidaemia, and offering insights for future research directions and clinical treatment strategies.

## 2 Mechanisms of gut flora affecting hyperlipidaemia

According to the current research, the absorption and metabolic decomposition mechanisms of lipids by gut flora can be broadly classified into three types: gut flora can regulate lipids through short-chain fatty acids (Ren et al., 2023) bile acids, bile acids, bile acids, bile acids, bile acids, bile acids, bile acids, bile acids, bile acids, and bile acids (Duan et al., 2021) and the angiopoietin-like family of proteins (Landfors et al., 2023). Other mechanisms such as the regulation of gut lipids by oxidized trimethylamine other products of gut flora, such as trimethylamine oxide, also have an impact on lipid metabolism in the body (Yoo et al., 2021).

### 2.1 Regulation of organismal lipids by bile acids (BAs)

Bile acids not only regulate the metabolism of cholesterol and fat-soluble vitamins, but also the structural composition of microbiota in the gut. Gut microbiota influence lipid regulation by affecting the whole process of bile acid metabolism. ① De-conjugation: A large number of studies have pointed out that gut microbiota, such as Lactobacillus and Bifidobacterium, have been detected to regulate the de-conjugation of conjugated bilirubin with bile salt hydrolase (BSH), which is required for the de-conjugation of conjugated bilirubin, and that BSH produced by Lactobacillus catalyzes the conversion of glycine- and taurine-bound BAs to unconjugated BAs, BSH produced by Lactobacillus can catalyze the conversion of BAs bound to glycine or taurine to unbound BAs (Zhou et al., 2023). ② 7-Dehydroxylation (recombinant cytochrome P450 7A1, CYP7A1): 7-Dehydroxylation of bile acids by gut microbiota is one of the most important metabolic transformations, which can regulate the production of secondary bile acids, and 7-Dehydroxylation is mainly found in Eubacterium and Clostridium (Hyemin and Yoon, 2024). ③ Oxidation and differential isomerisation: Recent studies have shown that both Eubacterium and Clostridium are capable of producing hydroxysteroid dehydrogenase (HSDH), which is capable of carrying out reversible redox and hydroxyl epoxidation of bile acids, leading to differential isomerisation (Yu et al., 2023). ④ Recent studies have shown that microbiota can also couple bile acids through the addition of a variety of amino acids other than glycine and taurine, and that the coupling of bile acids to amino acids is mediated by the activity of bile salt hydrolases (BSH), previously unknown acyltransferases, and that these BSH further expand the diversity of bile acids, and that this enzyme can essentially act in reverse to produce many microbially conjugated bile acids (MCBA), while MCBA production was widespread among gut bacteria, thus greatly increasing the diversity of bile acids and their roles (Guzior et al., 2024). Microbial coupling of bile acids: recent studies show that gut flora can also bind a small number of amino acids to free bile acids. ⑤ Esterification: *In vitro* studies have found that sulfate esterase activity has been detected in Clostridium spp., Fusobacterium spp., and Peptococcus spp. (Camilleri et al., 2023). These enzymes and reactions regulate lipid metabolism.

### 2.2 The role of short chain fatty acids (SCFAs) in the regulation of lipids in the body

The gut flora are involved in participating in the production of short chain fatty acids (SCFAs), with acetic acid, propionic acid, butyric acid and their salts as the main products. Acetic acid is produced by Bacteroides spp., Bifidobacterium spp., and Ruminococcus spp.; propionic acid by Clostridium spp.; and butyric acid by Bacteroides and Clostridium spp. (Sant’ Ana et al., 2024). Propionic acid has been shown to be able to lower the body temperature. It has been shown that propionic acid can reduce cholesterol and fatty acid production in the body and butyric acid can enhance the uptake of cholesterol by the liver by attenuating the synthesis of cholesterol in the liver, thus further transferring cholesterol in the blood to the liver, thus lowering the concentration of cholesterol in the blood (Zeng et al., 2023). The short chain fatty acids (SCFAs) can also activate the liver’s ability to synthesize cholesterol. Meanwhile, SCFAs can activate hepatic adenosine monophosphate-activated protein kinase (AMPK)/adenosine cyclophosphate (cAMP)/protein kinase A (PKA). The response elements, such as AMPK/adenosine cyclophosphate (cAMP)/protein kinase A (PKA)/peroxisome proliferators-activated receptors (PPARs), bind to the protein pathway to increase oxidative metabolism and reduce lipogenesis, and on the other hand inhibit the synthesis of cholesterol in the liver (Li et al., 2021). The effect of PPARs on cholesterol synthesis in the liver is also inhibited.

### 2.3 Regulation of organismal lipids by the angiopoietin-like proteins (ANGPTLs) family

**Figure 1:**
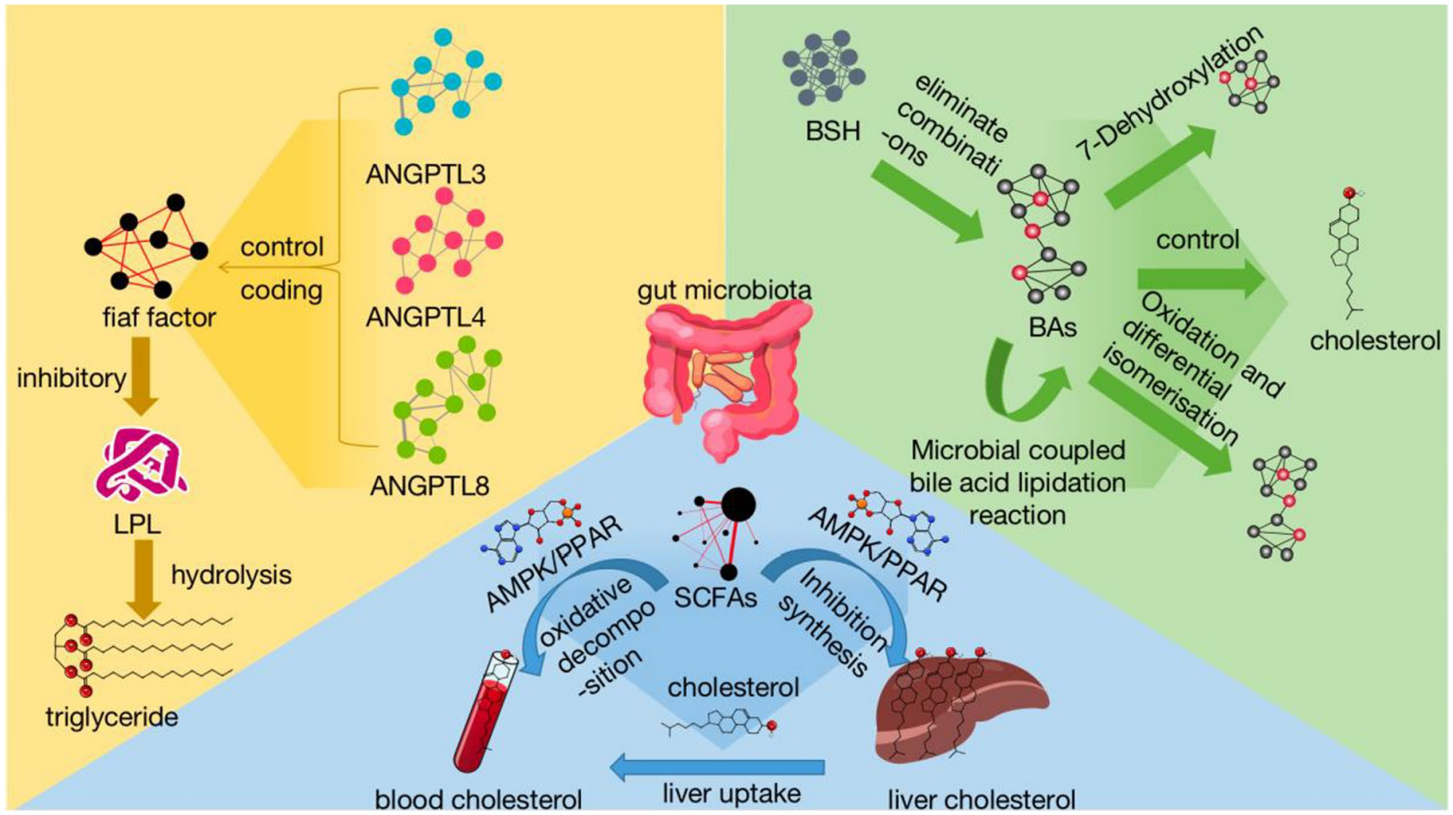
Mechanisms of gut microbial regulation of lipids reflects the influence of gut microbiota on hyperlipidemia in various treatment methods. Among them, gut microbiota regulates BAs from several aspects: dissociation, 7-dehydroxylation reaction, oxidation and isomerization, and microbial coupling of bile acids. The gut microbiota increases cholesterol utilization in the blood through pathways such as AMPK/PPAR by affecting the generation of SCFAs, while inhibiting liver cholesterol synthesis. Intestinal microbiota can inhibit LPL and regulate dyslipidemia by regulating fiaf factors and ANGPTL 3, 4, 8. SCFAs, short-chain fatty acids AMPK signaling pathway:adenosine monophosphate-dependent protein kinase-mediated signaling pathway PPAR signaling pathway:peroxisome proliferator-activated receptor-mediated signaling pathway; BAs, bile acids; BSH, bile salt hydrolases; ANGPTL, angiotensin proteins fiaf factor:fasting-induced adipokines; LPL, lipoprotein lipase.

Angiopoietin-like proteins (ANGPTL3, ANGPTL4, ANGPTL8) are the main family of angiopoietin-like proteins, which mainly regulate the fasting-induced adipocyte factor (Fiaf), which encodes an inhibitor of lipoprotein lipase (LPL). LPL hydrolyses triglycerides, and the Fiaf factor controls lipids by inhibiting triglyceride metabolism (Landfors et al., 2023). Fiaf factor controls blood lipids by inhibiting triglyceride metabolism. It has been shown that the gut flora of Lactobacillus, *Bacteroides thetaiotaomicron* and *Enterococcus faecalis* can regulate the expression of Fiaf factor and increase its expression. Meanwhile, Angiopoietin-like proteins3 (ANGPTL3) and HDL-C levels are positively correlated, ANGPTL3 is mainly expressed in the liver and can inhibit the enzymatic activity of LPL and EL, thereby suppressing the hydrolysis of triglycerides (TG), leading to an increase in the levels of TG rich lipoproteins (such as VLDL and chylomicrons) in plasma (Gugliucci, 2024). Angiopoietin-like proteins4 (ANGPTL4) is positively correlated with TG levels, and the ANGPTL4 protein inhibits the formation of LPL dimers, which are converted to inactive monomers and inhibit their activity. ANGPTL4 is upregulated during fasting or exercise, which inhibits LPL activity and reduces LPL activity in WAT, allowing TG to enter peripheral tissues and thus increasing TG levels in the blood (Song et al., 2023). Angiopoietin-like proteins8 (ANGPTL8) is positively correlated with TG, ANGPTL8 is upregulated under feeding conditions, forming a complex with ANGPTL3 to inhibit LPL activity in the circulatory system and peripheral tissues, thereby increasing TG levels in the blood. In addition, blood glucose levels are closely related to circulating ANGPTL8 concentration, and blood glucose is closely related to blood lipids, thereby further affecting blood lipids (Ye et al., 2023). In addition, the Angiopoietin-like proteins (ANGPTL) family of proteins has the potential to become an important locus for the treatment of hyperlipidaemia and diabetes in the future (Figure 1).

## 3 Hyperlipidaemia treatment and gut microbiota

Currently, hyperlipidaemia is treated with a variety of treatments broadly classified as: modern drug therapy, natural Chinese medicine and compound preparation therapy, biological therapy, exercise and diet therapy. All of these treatments treat hyperlipidaemia by regulating lipid metabolism in the body and regulating gut microbiota.

### 3.1 Modern medication

#### 3.1.1 Statin

Numerous studies have shown that the lipid-regulating mechanism of statins mainly lies in the competitive inhibition of the rate-limiting enzyme HMG-CoA reductase in the cholesterol synthesis pathway, in addition to its ability not only to cause a significant enhancement of the expression level of the LDL-C receptor in the blood, which can produce an inhibition of the synthesis of hepatic LDL-C, but also to promote the plasma LDL -C clearance, contributing to a decrease in TC concentrations in hyperlipidaemic patients (Liu et al., 2023). According to Wang Lijun team research shows that after taking statin drugs in the blood lipid normal standard population and the blood lipid is still abnormal people’s gut flora of the test results show that the standard group of patients with gut flora diversity is significantly higher than that of the blood lipid is still abnormal group of patients, in which the two groups of patients in the gut flora of the main differences in the flora of the bacteria have 10 bacterial species (Lijun et al., 2021). According to the study showed that the subdoligranulm and *Akkermansia muciniphila* and lipid metabolism correlation is greater, both can be through the fermentation reaction to produce short-chain fatty acids (SCFAs), rare micrococcus genus abundance of reduced levels of butyrate can be resulted in the main component of the SCFAs reduced levels of butyrate can reduce the synthesis of cholesterol in the liver, and at the same time, butyrate can reduce the cholesterol synthesis in the liver, butyrate can also be used as the major component of the short chain fatty acids (SCFAs). Butyrate is able to reduce the synthesis of cholesterol in the liver, and at the same time increase the uptake of cholesterol in the blood by the liver, thus transferring the cholesterol in the blood to the liver, and further reducing the concentration of cholesterol in the blood, while mucinophilic Acleromyces spp. are related to the gut flora product trimethylamine n-oxide (TMAO), which is able to inhibit the synthesis of bile acids and their transfer, leading to a reduction in the reverse transport of cholesterol. TMAO can inhibit the synthesis and transport of bile acids, leading to the reduction of reverse cholesterol transport, thus affecting the lipid metabolism *in vivo* (Lijun et al., 2021; Peng et al., 2023). Trimethylamine n-oxide (TMAO) can affect lipid metabolism in the body by inhibiting bile acid synthesis and transport, leading to a reduction in reverse cholesterol transport. Another study showed that after simvastatin treatment, patients’ TC, TG, LDL-C levels were significantly reduced, HDL-C levels were greatly increased, and the proportion of dominant bacteria such as Lactobacillus and Bifidobacterium in the gut flora was significantly increased; the proportion of nondominant bacteria such as Firmicutes and Enterococcus was greatly decreased, and the abundance of Gram-positive bacteria was significantly increased. The abundance of Gram-positive bacteria increased significantly (Peng et al., 2023). This suggests that simvastatin, while lowering lipid levels in patients with hypercholesterolaemia, also produces a slight anti-inflammatory effect modulating the composition of the gut microbiota, resulting in a significant increase in the proportion of probiotics. Simvastatin can maintain the dynamic balance between the gut microbiota and blood lipids to achieve better lipid regulation and control (Xu et al., 2023). In addition, the LPS-TLR4-Myd88 mechanism is also an important pathway for statins to lower blood lipids. Research has found that the TLR4-Myd88 signaling pathway is associated with lipid metabolism, and TLR4 is considered a potential receptor for LPS. LPS translocation causes abnormal cholesterol metabolism and elevated serum LDL-C levels via TLR4-Myd88 in hepatocytes. Statins can inhibit the expression of TLR4 on the cell membrane of liver cells and intestinal epithelial cells, and regulate the TLR4-Myd88 signaling pathway. The regulation of gut microbiota can effectively reduce LPS levels and control lipid metabolism. At the same time, the TLR4-Myd88 pathway is restricted by SCFA, and it has been previously found that statins can increase the production of SCFA in intestinal bacteria. The butyrate produced by intestinal bacteria can reduce the expression of TLR4 and Myd88 in the liver of mice, thereby reducing abnormal blood lipid elevation (Wang et al., 2022). Statins are now used as the first-line drugs for the treatment of hyperlipidaemia, with relatively small side effects on the human body, lipid-lowering efficacy is more accurate, and at the same time, according to the above research shows that it has a benign change in the structure of the gut flora, and it is a class of lipid-regulating drugs that are widely used in clinical practice at the present time.

#### 3.1.2 Fibrates

Fenofibrate is one of the most commonly used lipid-lowering drugs in the clinical first-line use of drugs for the treatment of hyperlipidaemia and related cardiovascular diseases. Fenofibrate has good effect in lowering TG and correcting TC metabolic disorders, and it can significantly increase peroxisome proliferator-activated receptor *γ* (PPARγ) and sterol Reg- Ulatory Element Binding Protein-1c (SERB1c) in the liver. Ulatory Element Binding Protein-1c (SREBP-1c) proteins were expressed to regulate the lipid levels of Zhang et al. (2020). Studies have shown that beta-agonists regulate blood lipid levels. It has been shown that while regulating blood lipids, fibrates increase the relative abundance of bacteroides and decrease the relative abundance of firmicutes, and it is now widely accepted that obesity and obesity-related physiological indicators are related to the ratio of firmicutes and bacteroides, so that betacyclics can have an impact on lipids *in vivo* by affecting the ratio of firmicutes and bacteroides. Another study also showed that the proportion of Firmicutes in the fecal microbiota of patients with fatty liver and hyperlipidaemia is higher, while the proportion of Bacteroidetes is lower. It also proves that the ratio of Firmicutes to Bacteroidetes is severely imbalanced during the pathogenesis of hyperlipidaemia. In addition, fenofibrate increases the abundance of Lactobacillus in the gut (Sun et al., 2022; Zhang et al., 2022). The increased abundance of Lactobacillus promotes the expression of peroxisome proliferator-activated receptors *α* (PPARα) to reduce lipid levels in the body (Behling et al., 2024). Fenofibrate has been shown to have a favorable effect. Meanwhile, although fenofibrate has a good effect of regulating blood lipid levels, long-term use of fenofibrate still has a certain degree of hepatotoxicity, which is manifested in the decline of its aspartate aminotransferase (AST) index, and the rise of its alanine transaminase (ALT), fenofibrate produces large amounts of deoxycholic acid (deoxycholic acid, ALT) to reduce the body’s lipid levels, which is the most important factor in the development of fatty liver. Fenofibrate causes bile acid toxicity through the production of large amounts of deoxycholic acid (DCA), which destroys hepatocytes and, in severe cases, further causes cirrhosis (Li et al., 2018). Fenofibrate has been shown to cause bile acid toxicity by producing high levels of deoxycholic acid (DCA), which can damage liver cells and in severe cases cause cirrhosis. The above studies show that as a commonly used lipid-regulating drug in clinical practice, fenofibrate can effectively lower blood lipids, regulate the ratio of gut flora and improve gut microbiota, but because it will produce a certain degree of hepatotoxicity to the human body, destroying hepatocytes, so it is necessary to pay attention to the dosage of the drug when used in the long term.

#### 3.1.3 Niacin

Niacin (nicotinic acid, NA), also known as vitamin B3, can effectively reduce TG, LDL-C, lipoprotein a [Lp(a)] levels, and increase HDL-C levels, and is commonly used clinically in combination with statins (Cai et al., 2022). Due to its dermal toxicity and itchy, red and swollen skin, acipimox, a nicotinic acid derivative with fewer side effects, is now mainly used, which is able to inhibit the release of free fatty acids from systemic adipose tissue and increase the plasma level of HDL-C levels in plasma to regulate blood lipids (Holeček and Vodeničarovová, 2020). Axiolimus regulates blood lipids by binding to the G coupled receptor 109A (GPR109A), while inhibiting the release of free fatty acids from adipose tissue throughout the body and increasing plasma HDL-C levels. Acipimox regulates silent mating type information regulation 2 homolog- 1 (SIRT1) by increasing the level of its derivative nicotinamide adenine dinucleotide (NAD+). On bile acid concentration, thereby promoting the multiplication and growth of gut Gram-positive bacteria (probiotics such as lactobacilli) and decreasing the proportion of Gram-negative bacteria (pathogens such as *Escherichia coli* or Salmonella), thus altering the structure of the gut flora (Tuteja, 2019). Lactic acid bacteria play an important regulatory role in lipid metabolism, which can inhibit the absorption of cholesterol in the body and lower blood lipids by inhibiting the expression of niemann-PickC1-likeprotein1 (NPC1L1), promoting the expression of ATP-binding cassette sub-family G member 5 (ABCG5) and recombinant cytochrome P450 7A1 (CYP7A1), and producing bile salt hydrolases (BSH) during the metabolism of lactic acid bacteria (Tuteja, 2019). Research has shown that niacin can also be produced in the metabolism of gut microbiota, through the indoleamine 2,3-dioxygenase (IDO) and kynurenine pathways produced by tryptophan. The metabolic products of gut microbiota can significantly reduce TG by directly binding to the nicotinic acid receptor GPR109A (Wu et al., 2023). However, niacin-based lipid-modulating drugs are limited to However, niacin-based lipid-regulating drugs are limited to their side effects, and their clinical use is relatively rare. How to improve or maintain the efficacy of lipid-regulating drugs while reducing the side effects is a major direction for future research.

### 3.2 Chinese natural medicine and compound preparation treatment

Before discussing traditional natural medicines, it is important to note that natural medicine must be practiced with rigorously tested and validated substances to ensure safety and effectiveness. Our research also follows this principle.

#### 3.2.1 Monascus purpureus went (Monascus/red yeast rice)

The main components of the erythropoietin class of drugs that produce lipid-lowering are the secondary metabolites Monacolin K or Lovastatin, Coenzyme Q10 (CoQ10), etc. (Cao et al., 2021). The administration of red yeast and its derivatives has been shown to reduce serum TG, TC and LDL-C concentrations, increase HDL-C concentrations, and improve cellular respiration, which is a significant effect in the treatment of hyperlipidaemia (Caligiuri, 2020). The effect of red yeast rice on the treatment of hyperlipidaemia is significant. Studies have shown that red yeast rice can improve the composition of the gut flora of mice on a high-fat diet by increasing the relative abundance of mucinophilic Ackermannia spp. (Xu et al., 2022). The study showed that red yeast rice could improve the composition of gut flora in mice on a high-fat diet by increasing the relative abundance of Ackermannia spp. Meanwhile, red yeast rice contributed to the increase of Verrumcomicrobiota spp. in the gut tract of mice fed a high-fat diet, while inhibiting Anaplasma spp. While both mucinophilic Akebia spp. and Verrumcomicrobiota spp. were able to regulate lipid levels by affecting the metabolism of short chain fatty acids (SCFAs) and trimethylamine n-oxide (TAMO) (Caligiuri, 2020; Lijun et al., 2021). SCFAs mediated activation of g-protein coupled Receptor 43 (GPR43) was able to reduce intracellular lipid spillage, while butyrate inhibited lipolysis of 3 T3-L1 preadipocytes through activation of GPR43, and butyrate inhibited lipolysis of 3 T3-L1 preadipocytes through activation of g-protein coupled Receptor 41 (GPR41) to inhibit lipolysis, and butyrate to inhibit the rise of blood lipid level, to achieve the effect of inhibiting the increase of blood lipid levels in the body (Peng et al., 2023). Another study also showed that red yeast synbiotics can reduce the number of *E. coli* and Salmonella and increase the number of Bifidobacterium and Lactobacillus, indicating that red yeast synbiotics can directly inhibit the reproduction of harmful bacteria, while *E. coli* and other flora can affect cholesterol and lipid metabolism through the production of trimethylamine n-oxide (TAMO), which affects the key enzymes for the synthesis of bile acids, such as recombinant cytochrome P450 7A1 (CYP7A1), and TMAO can reduce the conversion of cholesterol to bile acids to inhibit the metabolism of cholesterol and lipid metabolism. TMAO can also affect lipid metabolism and cholesterol homeostasis by reducing the conversion of cholesterol to bile acids. Trimethylamine n-oxide (TMAO) can also affect lipid metabolism and cholesterol homeostasis by reducing the conversion of cholesterol to bile acids (Cao et al., 2021; Yoo et al., 2021). In addition, it was found through experiments that red yeast caused a series of effects on the changes in Coriobacteriaceae UCG-002 and Lactococcus, reducing their quantity and proportion in the gut microbiota. The relative abundance of Coriobacteriaceae_UCG-002 was positively correlated with the hepatic mRNA expression of Proprotein Convertase Subtilisin/Kexin Type 9‌ (PCSK9) and inversely correlated with the intestinal mRNA expression of ATP-binding cassette transporter A1 (ABCA1) and the relative abundance of Lactococcus was inversely correlated with the hepatic mRNA expression of recombinant cytochrome P450 7A1 (CYP7A1). PCSK9 can bind to the low-density lipoprotein receptor (LDLR) on the surface of liver cells, form a complex, and be degraded inside the cells, resulting in a decrease in the number of LDLR. LDLR can bind to and clear LDL-C in plasma, so an increase in Proprotein Convertase Subtilisin/Kexin Type 9 (PCSK9) will lead to a decrease in LDL-C clearance, thereby increasing plasma LDL-C levels (Yang et al., 2021). Therefore, this indicates that red yeast can effectively reverse and improve the problem of imbalanced gut microbiota caused by hyperlipidaemia, and gut microbiota plays an important role in the treatment of hyperlipidaemia by directly or indirectly regulating lipid metabolism through various mechanisms. The red yeast rice have a wide range of effects, and the clinical use of lipid regulation efficacy is accurate, its side effects are small, the effect is relatively mild, and can positively regulate the gut micro-ecology, and has become the main drugs for the treatment of hyperlipidaemia in proprietary Chinese medicines, such as xuezhikang, zhibituo, zhibitai, and so on.

#### 3.2.2 Gynostemma pentaphyllum

Regarding the studies on the lipid-lowering effect of gynostemma pentaphyllum, nowadays, the extracts of active ingredients (saponins, polysaccharides and flavonoids, etc.) of gynostemma pentaphyllum are mainly used as gibberellin-like drugs (Cicero et al., 2021) gynostemma pentaphyllum is able to produce some antioxidant effects and reduce the level of lipid peroxidation *in vivo* while significantly lowering the levels of TC and LDL-C, TG (Zhou et al., 2023). In terms of its effect on gut microbiota, gynostemma pentaphyllum can reverse the increase in the number of Firmicutes and the decrease in the number of Bacteroidetes and Clostridium in the gut microbiota caused by hyperlipidaemia. Thereby increasing the abundance of Clostridium XIVa and Prevotella, and reducing the abundance of Clostridium XI (Cicero et al., 2021), achieving a significant reduction in the ratio of gut Firmicutes to Bacteroidetes in hyperlipidaemic rats, which can regulate the expression of short chain fatty acids (SCFAs) thereby altering lipid metabolism *in vivo*. It also increased the abundance of *Parabacteroides distasonis*, which produces a better anti-inflammatory effect (Zhou et al., 2023). Another study also showed that gynostemma pentaphyllum derivatives were able to significantly increase the number of probiotics such as Lactobacillus bifidus in the gut tract and significantly decrease the number of *Escherichia coli* in the gut tract, and the structure of the gut microbiota was effectively improved, while gynostemma pentaphyllum improves lipid metabolism by regulating gut microbiota and the toll-like receptor 2 (TLR2)/Recombinant NLR Family, Pyrin Domain Containing Protein 3 (NLRP3) signaling pathway, among which the TLR2/NLRP3 signaling pathway can regulate lipid metabolism by affecting proliferators-activated receptor *γ* (PPARγ) (Huang et al., 2018). At the same time, gynostemma pentaphyllum and gut probiotics have a synergistic effect to jointly regulate lipid metabolism (Zhu et al., 2023). In addition, studies have shown that taking metabolites of Gynostemma pentaphyllum can regulate the proportion of gut microbiota, leading to an increase in the production of tauro—*β*-muricholic acid (Tβ MCA) in the intestine. TβMCA induces the production and secretion of Glucagon-Like Peptide-1 (GLP-1) by reducing the transcriptional activity of the nuclear receptor farnesol X receptor (FXR), which primarily functions through a complex vagal endocrine mechanism (Yue et al., 2022). This proves that the metabolites of Gynostemma pentaphyllum can improve hyperlipidaemia through the FXR/GLP-1 pathway by reshaping the gut microbiota (Shu et al., 2022). The effects of gynostemma pentaphyllum on the metabolism of lipid metabolism gynostemma pentaphyllum drugs have a certain efficacy on metabolic diseases, its treatment of hyperlipidaemia related research is relatively small, but the clinical use of view, there is a clear regulation of blood lipids. Therefore, it is necessary to follow up the study of gynostemma pentaphyllum drugs on hyperlipidaemia.

#### 3.2.3 Astragalus

The components of Astragalus that produce effects on lipids are mainly astragalus total saponins and astragalus polysaccharides. Astragaloside regulates lipid metabolism mainly through direct action (Hoffman et al., 2022). Astragali polysaccharides regulate lipid metabolism mainly through a series of lipid metabolizing enzymes, such as recombinant cytochrome P450 7A1 (CYP7A1) and other pathways (Xie et al., 2020). Astragali Studies have shown that TC, TG and LDL-C levels decreased significantly in hyperlipidaemic model rats after administration of astragalus soup (Wang et al., 2022). The mechanism is that astragalus soup inhibits the expression of niemann-PickC1-likeprotein1 (NPC1L1) mRNA in the small intestine of hyperlipidaemic rats and promotes the expression of ATP-binding cassette sub-family G member 5/8 (ABCG5/G8) mRNA in the small intestine and up-regulates the phosphorylation of adenosine monophosphate-activated protein kinase (AMPK), inhibits the expression pf phosphorylation of phospho mitogen activated protein kinase (p- MAPK) and tumor necrosis factor-*α* (TNF-α) to promote the metabolism of TC (Wang et al., 2022; Xu et al., 2022). Meanwhile, the combined effect of Astragalus and gut flora reduced the expression of gut farnesoid X receptor (FXR) and fibroblast growth factor 15 (FGF15) in the ileum, thus leading to the reduction of recombinant cytochrome P450 7A1 (CYP7A1) and recombinant cytochrome P450 7B1 (CYP7B1), both of which are key enzymes in bile acid metabolism, thus affecting lipid metabolism (Xu et al., 2022). Another experiment also showed that the increase in the ratio of bacteroides to Firmicutes smegmatis in the gut tract of rats after the administration of astragalus polysaccharide could directly regulate the metabolism of dietary fiber by gut flora, and increase the concentration of short chain fatty acids (SCFAs), which are directly involved in the regulation of body lipids (Fu et al., 2022). Astragalus is often used in clinical applications to Replenish qi. Nowadays, it has been found in both clinical use and basic experimental research that Astragalus has the effect of regulating blood lipids, and has multiple mechanisms to positively regulate abnormal blood lipids in the gut microbiota. Therefore, it can be used as a type of traditional Chinese medicine to treat hyperlipidaemia.

#### 3.2.4 Reynoutria japonica

The extracts of Reynoutria japonica contain a variety of active ingredients such as scleroside, resveratrol, rhodopsin, etc. (Zhuang et al., 2021). Resveratrol is the most widely studied lipid-lowering agent. Among them, resveratrol has been studied most extensively for its hypolipidemic effect. Resveratrol achieves hypolipidemic effect by decreasing lipid synthesis and increasing adipic acid oxidation, and as a SIRT1 activator, it mainly controls lipid metabolism through adenosine monophosphate-activated protein kinase (AMPK) and peroxisome proliferators-activated receptor (PPAR) pathways (Wei et al., 2023). As a Silent information regulator of transcription 1 (SIRT1) activator, resveratrol regulates lipid metabolism mainly through the AMPK and PPAR pathways, and it can also activate the transcription of intrahepatic X receptor, which can encode the ATP-binding cassette transporter A1 (ABCA1), thus promoting HDL-C production and facilitating cholesterol production in blood, which is beneficial to cholesterol in blood. In addition, resveratrol is a transporter of ATP-binding cassette transporter A1 (ABCA1), which promotes the production of HDL-C and facilitates the reverse transport of cholesterol in the blood to reduce blood lipids (Wei et al., 2023; Tao et al., 2021). In addition, the autophagy of intrahepatic fat produced by resveratrol through the stimulation of SIRT1 (Silent information regulator of transcription 1)-FoxO1 (Forkhead box Transcription factor O1) pathway can effectively reduce TG and lipid droplets in hepatocytes, further reducing blood lipids. Meanwhile, the results of the study showed that resveratrol administered in the feed of hyperlipidaemic rats could significantly reduce the levels of TC, TG and LDL and increase the level of HDL (Zhuang et al., 2021). Resveratrol was found to regulate blood lipids in hyperlipidaemic rats. It was found that resveratrol could regulate the gut flora of rats, increase the proportion of probiotics and reduce the proportion of conditionally pathogenic bacteria, so that the structure of the gut flora tends to be normal, thus significantly increasing the activity of Fiaf factor in the liver of rats, and also lowering the levels of tumor necrosis factor-*α* (TNF-α), human macrophage chemoattractant protein-1 (MCP-1), malondialdehyde (MDA) and malondialdehyde (MDA), and also reducing the levels of TNF-*α*, MCP-1 and MDA. The activity of Fiaf factor in rat liver was also able to reduce the expression of TNF-α, human macrophage chemoattractant protein-1 (MCP-1), malonaldehyde (MDA), and increase the levels of superoxide dismutase (SOD) and total antioxidant capacity (T-AOC), which led to the reduction of low-level inflammation in the gut (Huang et al., 2018). Another study has shown that high-fat feeding is a good way to reduce low-level inflammation in the gut. Another study showed that the relative abundance of Bifidobacteria and Ackermannia increased and the relative abundance of Enterobacteriaceae such as Prevotella and Ruminocococaceae decreased after resveratrol was added to the diets of high-fat-fed rats, which also indicated that resveratrol has the effect of regulating the microecology of the gut tract (Huang et al., 2020). This also suggests that resveratrol has a role in regulating gut microecology. Reynoutria japonica has a wide range of efficacy, diuresis to remove yellowness, and also has good efficacy in the treatment of hyperlipidaemia, in which resveratrol is more prominent.

#### 3.2.5 Coptidis Rhizoma

Coptidis Rhizoma is rich in a variety of active chemical components, among which berberine, also known as berberine, as the most abundant and potent active ingredient, has been widely studied and clinically applied in the correction of lipid metabolism. Berberine promotes the expression of peroxisome proliferator activated receptor gamma co activator factor-1 *α* (PGC-1 α) by activating adenosine monophosphate-activated protein kinase (AMPK) and inhibits the expression of stearoyl-CoA desaturase-1 (SCD1), thereby reducing lipid accumulation and uptake. At the same time, it affects lipid metabolism in the body by promoting the expression of recombinant cytochrome P450 7A1 (CYP7A1) and inhibiting the expression of niemann-PickC1-likeprotein1 (NPC1L1), thereby promoting lipid clearance and consumption (Yin et al., 2020; Chen et al., 2016). The effect of berberine on lipid metabolism in the organism is shown in studies. Studies have shown that berberine can improve and treat hyperlipidaemia by correcting the abnormal ratio of gut flora in hyperlipidaemic rats, and its effects are mainly on Bacteroidetes and Firmicutes, suggesting that berberine can increase the abundance of Bacteroidetes and decrease the abundance of Firmicutes. The effect of berberine was mainly on the ratio of Firmicutes to Bacteroides was able to regulate the expression of short chain fatty acids (SCFAs) to influence lipid metabolism. Meanwhile, berberine caused a significant decrease in tumor necrosis factor-*α* (TNF-α) levels to promote TC metabolism and reduce inflammation (DiNicolantonio et al., 2022). Another study suggests that oral administration of berberberine can reduce the biosynthesis of Trimethylamine-N-oxide (TMAO) in the intestine, this action was performed by berberine’s metabolite dihydroberberine (a reductive berberine by nitroreductase in the gut microbiota), via a vitamine-like effect down-regulating Choline-Trimethylamine (TMA)-TMAO production pathway (Cao et al., 2019). As can be seen from the above, Coptidis Rhizoma is able to regulate lipid metabolism directly or indirectly through various mechanisms, so it is more often used as one of the drugs for the treatment of hyperlipidaemia in clinical practice.

#### 3.2.6 Chinese medicine compound preparation

Xuezhikang, Zhibitai and Zhibituo are commonly used in the treatment of hyperlipidaemia, and all of them contain red yeast and red yeast extract as main ingredients. Xuezhikang is a natural compound preparation made from indica rice inoculated with special red yeast, fermented and refined, which contains 13 kinds of natural compound statins and many other active ingredients (Duan et al., 2023). Zhibitai is mainly composed of red yeast, white atractylodes, Alisma, and hawthorn. The main active ingredients of this drug are Atractylenolide I in Atractylodes macrocephala, Alismatal in Alisma, Oleanolic acid and Ursolic acid in Hawthorn, and various natural compound statins in Monascus, all of which can regulate blood lipids, anti-inflammatory, and promote blood circulation to remove blood stasis (Ma et al., 2022). The main components of Zhibituo are red yeast, hawthorn, and Atractylodes macrocephala. Regulating blood lipids with effective ingredients such as natural statins, Atractylodes macrocephala, Oleanolic acid, Ursolic acid, etc. (Liang et al., 2019). As a natural compound contained in xuezhikang, zhibitai and zhibituo, red yeast is the main ingredient to regulate blood lipids. Red yeast regulates the activation of g-protein coupled Receptor 43 (GPR43) mediated by short chain fatty acids (SCFAs) by affecting the proportion of gut microbiota, thereby inhibiting lipolysis in 3T3-L1 preadipocytes and g-protein coupled Receptor 41 (GPR41) mediated by SCFAs. At the same time, gut microbiota regulates the metabolism of trimethylamine n-oxide (TAMO) and affects key enzymes such as recombinant cytochrome P450 7A1 (CYP7A1) for bile acid synthesis, thereby regulating cholesterol and lipid metabolism. Oleanolic acid and ursolic acid can correct the imbalance of gut flora by increasing the number of bifidobacteria and *Lactobacillus acidophilus* and decreasing the number of *Escherichia coli*, and reduce inflammatory factors such as TNF-alpha and lipopolysaccharide, thus regulating the level of blood lipids (Tingdina, 2019). Atractylenolide I affects the succinic acid pathway by upregulating the abundance of Bacteroidetes, leading to an increase in intestinal propionic acid content. Propionic acid can reduce cholesterol and hepatic fatty acid production in the body, thereby improving blood lipids (Shengyuan et al., 2017).

### 3.3 Biotherapy

Biological therapy for hyperlipidaemia is mainly divided into two categories: flora transplantation and microbiological agents. Bacterial colony transplantation is to isolate and transplant part of the fecal flora from normal human gut tract or part of the flora from human milk into patients with hyperlipidaemia, so as to achieve the purpose of adjusting the gut flora. Studies have shown that *Enterococcus faecalis* isolated from human milk or colostrum can absorb cholesterol to varying degrees, and its ability to lower cholesterol ranges from 25.2 to 64.1% (Xue et al., 2021). The ability to lower cholesterol ranged from 25.2 to 64.1%. For the treatment of hyperlipidaemia microbial preparations are now more bifidobacteria research, such as bifidobacteria triple capsules containing concentrated bifidobacteria, *Lactobacillus acidophilus* and *Enterococcus faecalis*, which in addition to harmful microbiota with effective antibacterial activity, it is also able to increase the proportion of beneficial bacteria, thus helping to improve the gut microbiota disorders, it can also increase the proportion of beneficial bacteria, thus helping to improve the disturbance of gut microbiota (Ren et al., 2021). Meanwhile, studies have shown that after 3 months of probiotic treatment, patients with dyslipidemia showed a significant increase in two lipid metabolites (acetylcarnitine and free carnitine), a significant decrease in TC, TG, and LDL-C levels, and a significant increase in HDL-C levels. On the other hand, probiotic intake does not affect the efficacy of other lipid-lowering drugs, such as lovastatin, and also slows down inflammatory reactions in the liver (Huang et al., 2021). In addition, supplementing the body with prebiotics is an indirect treatment method to improve the imbalance of gut microbiota. Supplementing with exogenous prebiotics and other organic substances can selectively promote the metabolism and proliferation of beneficial bacteria such as bifidobacteria and lactobacilli in the body, improve the gut microbiota environment, and indirectly regulate blood lipids (Zhang et al., 2023). The results of the above studies are consistent. The results of the above studies consistently show that microbial agents can improve the structure of gut microbiota to regulate blood lipid levels, with less adverse effects, and can be used in combination with other therapeutic modalities.

### 3.4 Exercise diet therapy

A sensible and healthy diet combined with moderate exercise can regulate blood lipids, mostly as an adjunct to other treatments. Reducing lipid intake is also the key to regulating blood lipids. A low-fat diet not only reduces the blood lipid level, but also normalizes the bacterial structure, reduces the number of non-beneficial bacteria in the intestine, increases the proportion of probiotics, enriches the abundance of gut flora, and reshapes the microbial structure of the intestine, whereas a prolonged high-fat diet promotes the catabolism and metabolism of *E. coli* to choline, thereby increasing the level of trimethylamine n-oxide (TMAO) in the body and further causing lipid metabolism disorders (Yoo et al., 2021). Meanwhile, it has been shown that dietary soluble dietary fiber can also induce changes in the gut microbiota by competing for gut cell attachment by commensal bacteria and reducing the reabsorption of BAs in the ileum as a major mechanism for lowering cholesterol, as well as preventing invasion of pathogens by regulating innate and direct defense mechanisms (Wang et al., 2023). Exercise can alter the structure of the gut microbiota and also produce lactic acid, which is absorbed by the microbiota and converted into short chain fatty acids (SCFAs) to participate in lipid metabolism. Lactic acid produced by exercise can be absorbed by microbiota and converted into SCFAs, which are involved in lipid metabolism (Ballan et al., 2020). Sports events inhibit insulin secretion, and after inhibiting insulin secretion, the body weakens the transcription of Angiopoietin-like 8 (ANGPTL8) in the liver through the Phosphatidylinositol 3-kinase (PI3K)/mammalian target of rapamycin (mTOR)/CCAAT enhancer binding protein alpha (CEBP *α*) pathway, reduces the binding of ANGPTL8 to Angiopoietin-like 3 (ANGPTL3) protein, increases lipoprotein lipase (LPL) activity, and especially increases the uptake of TG by the heart and skeletal muscles after meals, thereby exerting a regulatory effect on dyslipidemia. It also indicates that insulin plays an important role in regulating blood lipids (Holscher, 2020 and Table 1).

**Table 1:** Effect of each treatment modality on gut microbiota and side effects.

Treatment method	Main mechanisms of influence of gut microbiota	Side effects	Firmicutes/Bacteroidetes ratio	Gram-positive/negative bacteria ratio	Clinical relevance	References
Statin	SCFAs, BAs	Relatively small	Go down	Go up	For the study of these treatment methods, we can choose more effective ways to treat hyperlipidemia through their different mechanisms of action on gut microbiota in clinical applications. Alternatively, supplementing existing treatment methods with other main mechanisms can more efficiently treat hyperlipidemia.	Lijun et al. (2021), Peng et al. (2023), and Xu et al. (2023)
Fibrates	SCFAs, BAs	Hepatotoxicity, GI reactions				Zhang et al. (2022) and Behling et al. (2024)
Nicotinic acid	BAs	Erythema and itching of the skin				Cao et al. (2021) and Caligiuri (2020)
Red yeast rice	SCFAs	Relatively small				Xu et al. (2022), Yang et al. (2021), Zhou et al. (2023)
Gynostemma	SCFAs, BAs	Relatively small				Zhu et al. (2023), Yue et al. (2022), Hoffman et al. (2022), and Xie et al. (2020)
Astragalus	SCFAs, BAs	Relatively small				Xu et al. (2022), Fu et al. (2022), Zhuang et al. (2021), and Wei et al. (2023)
Reynoutria japonica	SCFAs, BAs	Relatively small				Huang et al. (2018), Huang et al. (2020), and Yin et al. (2020)
Coptidis Rhizoma	SCFAs, BAs	Relatively small				DiNicolantonio et al. (2022), Cao et al. (2019), Duan et al. (2021), and Ma et al. (2022)
Chinese medicine compound preparation	SCFAs, BAs	Relatively small				Xue et al. (2021)
Biotherapy	SCFAs, BAs	Relatively small				Zhang et al. (2023) and Ballan et al. (2020)
Exercise Diet Therapy	SCFAs, BAs, ANGPTL	Not have				Holscher (2020), Huang et al. (2022), and Liu et al. (2024)

SCFAs, short-chain fatty acids; BAs, bile acids; ANGPTL, angiotensin proteins.

At present, the combination of Chinese and Western medicine with the aid of exercise and diet therapy is a more effective treatment for hyperlipidaemia with relatively few side effects and better regulation of gut microecology. In summary, various treatment methods are closely related to gut microbiota, which affect the structure of gut microbiota, and the gut microbiota and drugs simultaneously regulate the abnormal lipid level of the organism, so as to achieve the purpose of treating hyperlipidaemia. Gut microbiota have become a new target for research and treatment of hyperlipidaemia and other metabolic diseases, which is of great significance.

## 4 Conclusion

In the latest research progress, there is an inseparable relationship between the treatment of hyperlipidaemia and gut microbiota, which plays an extremely important role in the treatment process. This review aims to describe the impact and mechanisms of gut microbiota on the treatment of hyperlipidaemia. We have summarized the effects of various treatments on gut microbiota production, studied modern chemical drugs, natural traditional medicines, biological therapies, and exercise and diet therapy. In these treatments, gut microbiota mainly affects hyperlipidaemia through the following mechanisms: (1) Intervention in bile acid synthesis, such as gut microbiota intervening in bile acid synthesis by controlling the metabolism of recombinant cytochrome P450 7A1 (CYP7A1) and trimethylamine n-oxide (TAMO), and regulating cholesterol metabolism through the fibroblast growth factor 15 (FGF15)-ATP-binding cassette sub-family G member 5/8 (ABCG5/G8) pathway by affecting farnesoid X receptor (FXR). (2) By regulating the synthesis of short chain fatty acids, such as the production of various short chain fatty acids by gut microbiota, it can directly affect the generation and uptake of cholesterol. At the same time, short chain fatty acids can regulate blood lipids in various ways through protein pathways such as activating adenosine monophosphate-activated protein kinase (AMPK), adenosine cyclophosphate (cAMP), and peroxisome proliferators-activated receptors (PPARs). (3) By regulating the metabolism of the angiopoietin like protein family, gut microbiota can directly affect LPL by controlling the production and metabolism of Angiopoietin-like 3,4,8 (ANGPTL3, 4, 8), thereby reducing blood lipids. (4) Regulating blood lipids through changes in the proportion of gut microbiota, such as affecting the ratio of Firmicutes and Bacteroidetes, can affect and reflect lipid metabolism levels. At the same time, various treatment methods also cause varying degrees of changes in the proportion of gut microbiota. The above summary can further deepen our understanding of the relationship between gut microbiota and the treatment of hyperlipidaemia, and provide new strategies for the diagnosis and treatment of diseases, as well as diversified and scientific treatments. At the same time, future research may provide important assistance in exploring the pathogenesis of hyperlipidaemia in terms of gut microbiota and the impact of other treatments for hyperlipidaemia.

## 5 Outlook

With the successful completion of microbiome sequencing, the relationship between gut microbiota and the development and prevention of diseases will be better understood and recognised. The study of gut microbiota will certainly become an important part of human healthcare in the future. In the past decade, the research on gut flora is only the tip of the iceberg, and we can foresee that in the near future, there will be an endless number of studies that will add to our understanding of the gut flora and its regulation of the metabolic homeostasis of the body, so that it can become a new therapeutic agent for the treatment of chronic metabolic diseases, such as hyperlipidaemia. Studies have shown that synergistic diagnosis and treatment of Chinese and Western medicine is particularly effective, and in the future clinical treatment of hyperlipidaemia it may be possible to use a multi-modality combination therapy. Through drug treatment combined with exercise diet therapy, microbial agents and other methods, so as to achieve the prevention of hyperlipidaemia, with a view to prevention and treatment can be combined to establish a solid insight into the prevention of hyperlipidaemia of the whole population barriers. In addition, it is worth studying and exploring whether gut microbiota and other metabolic diseases have similar mechanisms and effects during the onset and treatment process.
